# The Preterm Clinical Network (PCN) Database: a web-based systematic method of collecting data on the care of women at risk of preterm birth

**DOI:** 10.1186/s12884-018-1967-y

**Published:** 2018-08-17

**Authors:** Jenny Carter, Rachel M. Tribe, Jane Sandall, Andrew H. Shennan, the UK Preterm Clinical Network, Zarko Alfirevic, Christine Adamson, Phil Bennett, Elizabeth A. Bonney, Angharad G. Care, Manju Chandiramani, Sean Daly, Anna L. David, Helen Claire Francis, Ramesh Ganapathy, Joanna Girling, Natalie Greenwold, Natasha L. Hezelgrave, Catherine Hillman, Fatemeh Hoveyda, Catherine P. James, Matthew Jolly, Tony Kelly, Mani Malarselvi, Bradley N. Manktelow, Shalini Patni, Tara Selman, L. R. Shankar, Andrew Sharp, Nigel Simpson, Sarah J. Stock, Vasso Terzidou, Berrin Tezcan, Graham Tydeman, F. A. Vecsei

**Affiliations:** Department of Women and Children’s Health, School of Life Course Sciences, Faculty of Life Sciences and Medicine, King’s College London, London, UK

**Keywords:** Preterm birth, Clinical databases, Clinical registries, Clinical networks, Clinical audit

## Abstract

**Background:**

Despite much research effort, there is a paucity of conclusive evidence in the field of preterm birth prediction and prevention. The methods of monitoring and prevention strategies offered to women at risk vary considerably around the UK and depend on local maternity care provision. It is becoming increasingly recognised that this experience and knowledge, if captured on a larger scale, could be a utilized as a valuable source of evidence for others. The UK Preterm Clinical Network (UKPCN) was established with the aim of improving care and outcomes for women at risk of preterm birth through the sharing of a wealth of experience and knowledge, as well as the building of clinical and research collaboration. The design and development of a bespoke internet-based database was fundamental to achieving this aim.

**Method:**

Following consultation with UKPCN members and agreement on a minimal dataset, the Preterm Clinical Network (PCN) Database was constructed to collect data from women at risk of preterm birth and their children. Information Governance and research ethics committee approval was given for the storage of historical as well as prospectively collected data. Collaborating centres have instant access to their own records, while use of pooled data is governed by the PCN Database Access Committee. Applications are welcomed from UKPCN members and other established research groups. The results of investigations using the data are expected to provide insights into the effectiveness of current surveillance practices and preterm birth interventions on a national and international scale, as well as the generation of ideas for innovation and research. To date, 31 sites are registered as Data Collection Centres, four of which are outside the UK.

**Conclusion:**

This paper outlines the aims of the PCN Database along with the development process undertaken from the initial idea to live launch.

## Background

The UK Preterm Clinical Network (UKPCN), founded in 2013, is a network of doctors, midwives and researchers whose aim is to prevent the problems associated with preterm birth with emphasis on the antenatal surveillance of women at risk. However, there is a paucity of conclusive evidence on which to base this practice, with few national guidelines. The first UK National Institute for Health and Care Excellence (NICE) guideline on preterm birth was published only in 2015 [1]. In the absence of conclusive evidence, the methods of monitoring and preterm birth prevention strategies offered to women at risk vary considerably around the UK and depend on local maternity care provision [2].

A key aim of the UKPCN is to improve care and outcomes through the sharing of experience and knowledge, as well as building clinical and research collaborations. The network’s expertise could also be a utilized as a valuable source of evidence by others [3, 4]. The value of clinical networks and registries is increasingly recognized and they are particularly useful in areas where empirical evidence is lacking [5–7]. Where data is collected systematically, there is also potential for large scale bio-informatics studies and linkage with other datasets [8].

An initial resolution of the UKPCN was the systematic collection of standardized clinical data from UKPCN specialist preterm clinics. A bespoke database was required as no current clinical registries of this nature, in this field, were identified. In this paper, we describe the database development process from conception to live launch in December 2016. Posters describing this database were presented at the British Maternal & Fetal Medicine Society Annual meeting in Birmingham, UK, April 2016 [9] and at the 2nd European Spontaneous Preterm Birth Congress in Gothenburg, Sweden, May 2016 [10].

## Construction and content

### Main principles and scope

Prior to designing the database the UKPCN identified main principles, intended scope and key features. It was determined that the database should be designed to allow clinicians to easily audit their own practice, with an added facility to combine data for shared audit, service evaluation and research. In order to minimize the additional time burden for already busy clinicians, the design needed to cater for quick and easy entry of an agreed minimal dataset, including risk factors, surveillance methods, interventions and outcomes. The database also needed to allow for the addition of more detailed data, should it be required and when resources were available.

The vision was to collect data prospectively in preterm clinics, with outcomes added later. However, it was decided that the database should also have the flexibility to store historical data. This was considered important as many UKPCN preterm clinics were established and there was a wealth of data already collected for internal audit and service evaluation purposes. Combining these data could immediately provide a valuable source of material for investigation. Flexibility to accommodate data collection for small research projects not warranting a unique database was also considered to be an important component. Security, user-friendliness and accessibility were considered of vital importance to the project, and it was decided that an internet-based platform would be the most suitable.

### The platform and database schema

MedSciNet, a Swedish based company, were contracted to develop the application [11]. The Preterm Clinical Network (PCN) database and a web-based application to access the data was built using the MedSciNet Clinical Trial Framework (CTF), a self-contained environment that enables development, hosting, support and management of individual web-based solutions for clinical trials and studies, quality registries, medical biobanks and other required solutions within the field of academic medicine. The databases conform to relevant FDA, NIH and HL7 standards guidelines and recommendations. Microsoft.NET Framework and Microsoft SQL Server technologies were used for the platform development. A test database was created, piloted and refined after piloting and to incorporate adjustments recommended by the Research Ethics Committee. The data schema is described in Table 1.

**Table 1:** Database schema

Main forms (minimal dataset)				
Data form	Data level 1	Data level 2	Data level 3	Comments
Registration	Initials; date of birth; postcode; hospital number; NHS number; number of fetuses; consent to database; consent to storage of baby identifier			Initials, date of birth, hospital number and NHS number are transferred to separate “Patient Details Database”.
Clinic Record	Demographics	Expected date of delivery (EDD)		
		Gravida		Number of pregnancies, including current
		Parity		Number of live births or pregnancies ending at 24^+ 0^ or more weeks’ gestation
		Height (cm)		
		Weight (kg)		
		BMI (kg/m^2^)		Calculated from height and weight
		Age (at EDD)		Calculated from date of birth and EDD.
		Ethnicity		Drop down list
		Smoking status		Drop down list
		Lower super output area		Postcode converted to lower super output area.
	Risk factors/ reasons for referral	Previous preterm birth	Number of previous preterm births and earliest gestation	
		Previous premature ruptured membranes (PPROM)	Number of previous PPROMs and earliest gestation	
		Previous late miscarriage	Number of previous late miscarriages and latest gestation	
		Previous cervical surgery	Number of previous cervical surgeries and most significant procedure	
		Uterine abnormality		Drop down list
		Multiple pregnancy		Enter number
		Other risk factors		Drop down list and free text
	Preterm clinic visits	Date		Enter date
		Gestation		Calculated from date of visit and EDD
		Shortest cervical length		
		Fetal fibronectin results		
		Infection screen results		
		Other test results		
		Symptoms	None; abdominal/ back pain; tightenings; tightenings and pain; vaginal pressure; PV loss; other	
	Preterm birth interventions	Type	E.g. cerclage; progesterone; pessary; bedrest; admission	
		Sub-type	E.g. low vaginal cerclage; high vaginal cerclage; abdominal cerclage	
		Indication for intervention	History indicated; ultrasound indicated; emergency/rescue	
		Intervention date		
		Gestation		Calculated from EDD and date of intervention
		Date of delivery		
	Pregnancy outcome	Onset of labour	Spontaneous; induced; pre-labour caesarean	
		Gestation at delivery		Calculated from EDD and date of delivery
		Birthweight		
		Maternal outcomes	No significant morbidities; maternal infection or inflammation; pre-labour rupture of membranes; harm to mother from intervention; ITU admission Maternal death	
		Neonatal outcomes	Livebirth; stillbirth; miscarriage; NNU admission; infection (proven, ≤ 72 h); early neuro-developmental morbidity; late neuro-developmental morbidity; gastro-intestinal morbidity; respiratory morbidity; harm to infant from intervention; neonatal death	
Additional data forms	Comments *(for additional details if required)*			
Medical History	Tick and text boxes for recording of medical conditions and current medications.			
Obstetric History	Space for recording previous pregnancies: year; gestation, outcome; onset of labour; mode of delivery; preterm birth interventions; gestation at intervention.			
Cervical Surgery	Space for recording cervical surgery: year; type; anaesthetic; depth			
Antenatal details	Details of preterm interventions; day assessment episodes and antenatal inpatient nights, e.g. tocolysis; steroid administration; antibiotics; progesterone; cerclage; pessary.			
Delivery Details	Onset of labour; reason if not spontaneous; magnesium sulphate and antibiotics in labour; markers of maternal infection (e.g. pyrexia, test results); blood loss; date of discharge; number of postnatal nights.			
Neonatal Details	Date and time of delivery; gestation at delivery; date and time of rupture of membranes; mode of delivery; gender; birthweight; Apgar scores; congenital abnormalities; NNU admission; neonatal morbidities and death; discharge from hospital; number of inpatient nights (one form created per fetus).			
Research Participation Record	Record of patient’s participation in preterm birth research: study name; study ID; date of enrolment; study design; treatment allocation.			

### Information governance and ethical approval

Initially, permission was sought through NHS Trust Information Governance departments. However, there was no UK wide system in place for Information Governance regulation for the storage and sharing of clinical data and this proved to be time consuming and laborious. A decision was taken, therefore, to redefine the database as a “Research Database” and apply for Research Ethics Committee (REC) review through the UK Integrated Research Application System (REC Reference 16/ES/0093). REC approval is not mandatory for research databases in the UK, but the process allows for independent review of the protocol, procedures and security of the data, which is acceptable to NHS Trust Information Governance departments and future publishers of papers presenting the findings from projects investigating the data. Although local R & D department approval is not required by the REC, they are informed because they may wish to review their site’s capacity for collaborating in the project. They are also involved with the data sharing agreements which are required before the release of pooled data for approved research projects.

### The issue of data anonymity and patient consent

Prospective data collection, i.e. at the time of the clinic appointment, requires the recording of patient identifiers for later follow up of pregnancy outcomes. We were advised by our NHS Trust Information Governance department that written patient consent would be necessary because: a) the data would not be completely anonymous, and b) it is feasible to obtain consent from current patients. Obtaining patient consent also provides the opportunity to seek permission for storage of their baby’s NHS number and potentially long term follow up of the child.

Identifiable data has been kept to a minimum (i.e. initials, date of birth, hospital number, NHS number) and transferred, at registration, to a separate but linked Patient Details Database. On the main database, date of birth is converted to age (at expected date of delivery) and postcode converted to a marker of socio-economic status. Data collection centres outside the UK use alternative socio-economic indicators.

For historically collected data with known pregnancy outcomes Information Governance advised that patient consent would not be required. This was because historical data could be anonymised before transfer into the database, and because it would be impractical to consent women following discharge from maternity care.

### Data capture and flow

**Fig. 1:**
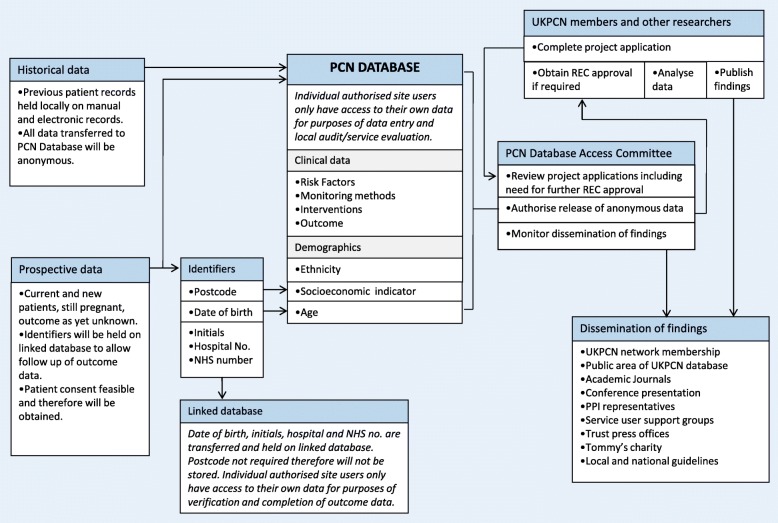
PCN database - data flow

**Fig. 2:**
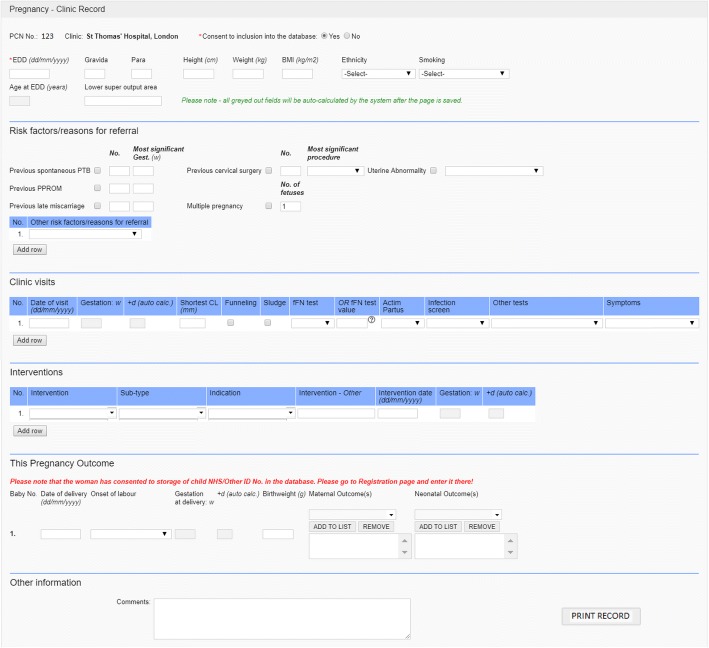
Clinic Record (minimal dataset)

Data are entered locally at Data Collection Centres under the supervision and direction of the local Lead collaborators. Figure 1 outlines the flow of data through the project. The single “Clinic Record” page captures the minimal dataset which includes demographics, referral criteria, surveillance methods, test results, preterm interventions and outcomes (Fig. 2). This record has been designed to include the COPOP core outcomes for preterm birth intervention studies [12]. Further information can be entered on additional, non-mandatory forms, e.g. detailed data on risk factors, medical and obstetric history, antenatal and delivery details, and other neonatal outcomes and health utilisation.

### Data management and quality control

The Project Lead, Project Manager and Database Access Committee Chair (named authors of this paper) are all based at the Department of Women and Children’s Health, School of Life Course Sciences, King’s College London, UK. The local lead collaborators are responsible for the delegation of data entry and monitoring duties to local site users, and for the preparation of their own historical data for transfer. The database has an inbuilt data monitoring facility, whereby locally appointed site monitors can monitor data, raise queries and “lock” data. Historical data is prepared for transfer and can be either entered manually, or imported via an Excel spreadsheet template.

### Funding

Funding for the initial design and development of the database was covered by an NHS Innovations Challenge Prize Fund, won by the Project Lead Professor Andrew Shennan in 2013 for his work in his preterm surveillance clinic. Tommy’s charity, who support the work of the Department of Women and Children’s Health, are contributing towards the ongoing support and maintenance.

## Utility and discussion

At present, UK hospital electronic patient records systems do not allow for the standardised and systematic collection of clinical data on women at risk of preterm birth. This is the first UK wide database or registry specifically designed to do so. At the time of Sharp and Alfirevic’s survey [2] of UK specialist preterm services, carried out in 2012 and 2013, only 23 of 210 NHS consultant led obstetric units provided a specialist preterm clinic. Current provision is unknown, but interest in this specialist field appears to be increasing, as reflected by the rise in membership of the UKPCN from 39 in 2013, to the current membership of 149 (March 2018). To date, 31 sites have registered as Data Collection Centres: these are predominately UK NHS hospital based, but also include hospitals in Australia, New Zealand, Republic of Ireland and Spain. Although many sites remain in set up 7 are already using the database and have consented 1020 women for prospective data collection (March 2018) with 94% of these also agreeing to long term follow up. Latest recruitment figures can be viewed on the website [15].

### Research projects using shared data and the database access committee

Local collaborators can investigate their own data freely, but researchers requiring access to data from other sites are required to submit an application to the Database Access Committee. The PCN Database Project Application Form allows applicants to provide details of their proposed project, and incorporates an “Applicant Agreement” and “Data Requested Form”. Applications are welcomed from UKPCN members and other established research groups. Projects may be based on a variety of study designs, for example: case-controlled studies designed to examine the relationship between risk factors or interventions and outcomes; cross sectional surveys; cohort studies and sample size calculations. The inclusion of COPOP core outcomes will facilitate comparison and combination of PCN data with other studies [13]. Project applications are reviewed by the Database Access Committee, which is comprised of members of the UKPCN, the RCOG Preterm Clinical Study Group [14], and the Guy’s and St Thomas’ Women’s Health Academic Centre’s preterm birth studies patient and public involvement (PPI) panel. The PCN Database Access Committee will scrutinize the quality of the proposal and academic team and will also consider whether further Research Ethics Committee approval is required.

### Long term follow up of child health

All specific research project timeframes are finite in nature, and most will have relatively short term follow up. Interventions used in pregnancy could have long term, as well as short term, effects on the child and could, potentially, persist into adulthood. A major advantage of the PCN Database is that it also acts as a registry of children who have been born to women at risk, who have undergone specialist preterm surveillance and who may have had preterm interventions, whether born prematurely or not. This offers researchers the possibility of investigating much longer term outcomes of the care of women attending preterm clinics. Patients are given the opportunity to opt in, or out, of longer term follow up, and are asked to indicate their specific consent to the storing of a unique identifier for the child’s health records (in the UK, the NHS number). Additional information collected on the children’s health beyond the initial neonatal outcomes will be determined according to the requirements of the proposed study. The PCN Database Access Committee review will include consideration of the need for further REC approval as well as the method of acquiring the necessary data, e.g. GP or other health records or linked databases, or direct approach to the mother (e.g. for questionnaire completion or face-to-face appointment for developmental assessment of the child). Investigators requesting data for follow up studies involving children beyond 16 years of age will be required to seek individual written consent of the said children. The children will be approached by the PCN Database project team via a communication sent to the most current address held by NHS care records.

### Future developments

Further developments will include using the database for data collection for small intervention studies, such as those managed by the Preterm Trials Consortium, a UK wide survey of maternity care provision for women at risk of preterm birth and patient experience studies. Other plans include future linkage with large population datasets so that comparisons can be made with the wider population, including low risk childbearing women.

Clinicians interested in registering as Data Collection Centres, both from the UK and outside, are invited to contact the corresponding author for more information.

### Database website

A public area of the website [15] serves as a source of information about the UK Preterm Clinical Network. It is used as a resource for the public, potential new UKPCN members, potential new Data Collection Centres and other health professionals. Useful links and publications resulting from projects using data from the database will be available on this part of the site.

## Conclusions

The PCN Database is an easy to use, secure, web-based facility for the storage of the clinical data of women at risk of preterm birth. It captures information about risk factors, specialist preterm surveillance, interventions and outcomes, and allows for the potential follow up of children for much longer than specific research projects usually permit. It is a valuable resource for both clinicians caring for women at risk of preterm birth and researchers investigating clinical care provision, current trends and planning future studies in this area.

## Availability and requirements

Project name: Preterm Clinical Network Database

Project home page: www.medscinet.net/ukpcn

Operating system(s): Windows Server 2012

Programming language: Microsoft SQL, C#, Java Script

Other requirements: none

License: no license required

## References

[1] National Institute for Health and Care Excellence (NICE) (2015). Preterm labour and birth: NICE Guideline [NG25].

[2] Sharp A, Alfirevic Z (2014). Provision and practice of specialist preterm labour clinics: a UK survey of practice. BJOG Int J Obstet Gynaecol.

[3] Hay MC, Weisner TS, Subramanian S, Duan N, Niedzinski EJ, Kravitz RL (2008). Harnessing experience: exploring the gap between evidence-based medicine and clinical practice. J Eval Clin Pract.

[4] Owtad P, Taichman R, Park JH, Yaibuathes S, Knapp J (2013). An idea for the future of dental research: a cloud-based clinical network and database. Journal of Curriculum and Teaching.

[5] Gliklich R, Dreyer N, Leavy M. Registries for evaluating patient outcomes: a User’s guide. Two volumes.(prepared by the outcome DEcIDE center [outcome sciences, Inc., a quintiles company] under contract no. 290 2005 00351 TO7.) AHRQ publication no. 13 (14)-EHC111. Rockville, MD: Agency for Healthcare Research and Quality. April 2014. Rockville, MD: Agency for Healthcare Research and Quality 2014.

[6] Workman TA. Engaging Patients in Information Sharing and Data Collection: The Role of Patient-Powered Registries and Research Networks [Internet]. Rockville (MD): Agency for Healthcare Research and Quality (US); 2013 Sep. Defining Patient Registries and Research Networks. https://www.ncbi.nlm.nih.gov/books/NBK164514 Accessed 14 Nov 2016. 24156118

[7] Baggi F, Mantegazza R, Antozzi C, Sanders D (2012). Patient registries: useful tools for clinical research in myasthenia gravis. Ann N Y Acad Sci.

[8] Bellazzi R, Diomidous M, Sarkar IN, Takabayashi K, Ziegler A, McCray AT (2011). Data analysis and data mining: current issues in biomedical informatics. Methods Inf Med.

[9] Carter J, Tribe RM, Shennan AH (2016). The UKPCN database: a standardised and systematic data collection method for preterm clinics (poster presentation BMFMS conference, 2016). BJOG Int J Obstet Gynaecol.

[10] Carter J, Tribe RM, Shennan AH. The Preterm Clinical Network Database: a standardised and systematic data collection method for preterm clinics. (poster presentation 2^nd^ European Spontaneous Preterm Birth conference, 2016), unpublished. 10.

[11] Medscinet website, available at: (http://www.medscinet.com) Accessed 31 July 2017.

[12] van ‘t Hooft J, Duffy JM, Daly M, Williamson PR, Meher S, Thom E (2016). A Core outcome set for evaluation of interventions to prevent preterm birth. Obstet Gynecol.

[13] Preterm Clinical Network Database. available at: http://www.medscinet.net/UKPCN Accessed 31 July 2017.

[14] Duffy JMN, Rolph R, Gale C, Hirsch M, Khan KS, Ziebland S, McManus RJ. And the international collaboration to harmonise outcomes in pre-eclampsia (iHOPE) (), Core outcome sets in Women's and newborn health: a systematic review. BJOG: Int J Obstet Gy. 10.1111/1471-0528.14694. 10.1111/1471-0528.14694 28421657

[15] Royal College of Obstetrics and Gynaecologists Preterm Labour Clinical Study Group, available at: (http://www.bmfms.org.uk/Preterm-birth-CSG/c-1-36.htm). Accessed 31 July 2017.

